# Exploring the Therapeutic Efficacy of Glioma Vaccines Based on Allo- and Syngeneic Antigens and Distinct Immunological Costimulation Activators

**DOI:** 10.4172/2155-9899.S5-004

**Published:** 2012-03-09

**Authors:** Apostolos Stathopoulos, Chrystel Pretto, Laurent Devillers, Denis Pierre, Florence M. Hofman, Alan L. Epstein, Hooman Farghadani, Carol A. Kruse, Martin R. Jadus, Thomas C. Chen, Virgil E.J.C. Schijns

**Affiliations:** 1 Department of Neurosurgery, Arlon Hospital, Arlon, Belgium; 2 Epitopoietic Research Corporation (ERC), Namur, Belgium; 3 Department of Pathology, University of Southern California, Keck School of Medicine, Los Angeles, California, USA; 4 Department of Neurosurgery, University of California, Los Angeles, California and the Jonsson Comprehensive Cancer Center, UCLA, Los Angeles, CA 90095, USA; 5 Veterans Affairs Medical Center, Long Beach, CA 90822, box 113, 5901 E7th St. and Chao Cancer Center, University of California, Irvine, Orange CA, USA; 6 Cell Biology & Immunology Group, Wageningen University, PO Box 338, 6700 AH Wageningen, The Netherlands; 7 Department of Neurosurgery, University of Southern California, Keck School of Medicine, Los Angeles, California, USA; 8 Department of General Surgery, Arlon Hospital, Arlon, Belgium

## Abstract

The efficacy of a various immunotherapeutic immunisation strategies for malignant glioma brain cancer was evaluated in the syngeneic CNS-1 Lewis rat glioma model. A prototype glioma cancer vaccine, which was composed of multivalent antigens derived from allogeneic and syngeneic cells and lysates, formed the prototype preparation of antigens. These antigens reflect the autologous antigens derived from the patient’s surgically removed tumor tissue, as well as allogeneic antigens form glioma tumor tissue surgically removed from donor patients. This antigen mixture provides a broad spectrum of tumor associated antigens (TAA) and helps to prevent escape of tumor immune surveillance when given as a vaccine. This antigen preparation was administered in a therapeutic setting with distinct single or multiple co-stimulation-favouring immunostimulants and evaluated for inhibition of tumor growth. Our prototype vaccine was able to arrest progression of tumor growth when co-delivered in a specific regimen together with the costimulating multi-TLR agonist, Bacille Calmette Guerin (BCG) and interleukin-2, or with the Toll-Like receptor (TLR) 7/8 activator resiquimod.

## Introduction

Glioblastoma multiforme (GBM) is an invasive malignant tumor of the central nervous system. Conventional therapy options include surgery, radiation, and chemotherapy, but with them the prognosis for GBM patients is limited to a mean survival time of only 14.6 months [1]. Immunotherapy is emerging as a novel complementary treatment option for a variety of malignancies including GBM. The use of successful passive immunotherapies based on the administration of immune elements, such as antibodies has proven very successful against various types of cancer. Well-known examples include antibodies that target tumor expressing receptors for epidermal growth factor (EGF), called Herceptin, those that target HER-2 [2], and those that are directed against angiogenic, tumor blood vessel growth-promoting vascular endothelial growth factor (VEGF), known as Avastin [3]. Recently, the antibody directed against an immune response inhibitory molecule, called cytotoxic T lymphocyte associated protein 4 (CTLA4), known as Yervoy®, has shown promising clinical efficacy in melanoma patients [4].

Apart from these passive antibody-based therapies, a range of active immunotherapies are in late stage development and are close to reaching approval as standard of care. These clinical studies clearly demonstrate that the immune system is able to discriminate cancer cells from normal cells following recognition of tumor associated antigens (TAA). Indeed a recent approval for a prostate cancer vaccine named Provenge®, was obtained from the FDA in April 2010, for the treatment of asymptomatic or minimally symptomatic metastatic, castrate-resistant (hormone-refractory) prostate cancer [5]. This recent approval has rejuvenated interest in the field as a whole.

In the present study we demonstrate a prototype brain cancer vaccine against gliomas which is composed of multivalent antigens derived from allogeneic and syngeneic cells and lysates. Our prototype is reflected in a clinical situation by autologous antigens derived from the patient’s surgically removed tumor tissue. In addition, glioma tumor tissue surgically removed from donor patients provides a second source of allogeneic antigens that can be isolated and subsequently stored for later use. This material provides a new source of TAA that may display HLA-restriction that may overlap with that on the patient’s glioma. They may serve to enhance the overall immune response. If processed under Good Manufacturing Conditions, it may provide an “off-the shelf” application. Relevant unique or shared TAAs overexpressed by tumor cells are present among thousands of irrelevant immunotolerant non-tumor associated antigens. The broad range of TAAs is preferred over vaccines with a mono- or oligo-valent antigenic content. These vaccines will prevent escape of tumor cells due to antigenic loss, or active MHC downregulation. In addition, a tumor antigen mixture also circumvents the use of monovalent synthetic peptides, which are restricted for use in patients with defined HLA types. By including TAAs of allogeneic origin, we additionally trigger allogeneic immune reactions. The haplotypes of CNS-1 and RG2 are fairly close, i.e., RT1^l^ vs RT1^lvl^, yet provide a mismatch that may be considered abberant self, and also induce an alloresponse. This may provide better protection due to the “non-self” immune recognition of these antigens.

Although allogeneic tumor antigens may provoke immune responses to non-self antigens in classical allogeneic immune rejections, glioma tumors, once established, are known to actively suppress the host’s immune system, by well characterized mechanisms [6], which often leads to subsequent evasion of immune surveillance. We therefore decided to test various signaling and costimulation-favouring immunostimulants in combination with our prototype vaccine antigen formulation for anti-tumor activity in an aggressive rat glioma brain tumor model, CNS-1, that is syngeneic in Lewis rats.

Here we demonstrate that our prototype vaccine is able to arrest progression of tumor growth when co-delivered in a specific regimen with the costimulatory-enhancing multi-TLR agonist, Bacille Calmette Guerin (BCG) adjuvant with interleukin-2, or with the TLR 7/8 activator resiquimod.

## Materials and Methods

### Tumor model

#### Glioma cells

Both CNS-1 tumor cells and RG2 (ATCC, CRL-2433) rat glioma cells were cultured in Dulbecco’s modified Eagle (DMEM) medium containing 10% bovine fetal serum (FBS), 5% antibiotics Penicillin, Streptomycin, and amphotericin B (Hyclone) in 175-mm^2^ flasks. The CNS-1 haplotype is RT1^l^ and RG2 is RT1^lvl^.

#### Animals

Rat CNS-1 cells (2 × 10^5^ cells/200 ul) were implanted subcutaneously (SC) using a 21 gauge needle into the right flank of 8-12 week-old (300 gram body weight) male Lewis rats. For each treatment group and control, 4-8 rats/group were used. All animal studies were approved by an independent ethical committee.

### Monitoring tumor growth

The sizes of the CNS-1 tumor volumes were measured using a caliper three times per week on Mondays, Wednesdays and Fridays to monitor the effects of each treatment group.

### Completion of experiment

Tumor implanted rats were sacrificed if they showed signs of discomfort, as defined by the ethical committee. For example if they appeared moribund due to weight loss, lethargy, ruffled fur, or when tumors showed ulceration. A mixture of Rompun and ketamine was used for anesthesia, followed by a dose of sodium pentobarbital for euthansia.

### Vaccine

The vaccine antigen preparation was composed of a mixture of haptenized CNS-1 cells and RG2 cells (1 × 10^e^6 CNS-1 syngeneic and 1 × 10^e^6 RG2 allogeneic glioma cells), together with lysates produced from 3 × 10^e^6 CNS-1 syngeneic cells and 3 × 10^e^6 allogeneic RG2 glioma cells. The haptinization method has been described before [7].

**Figure 1:**
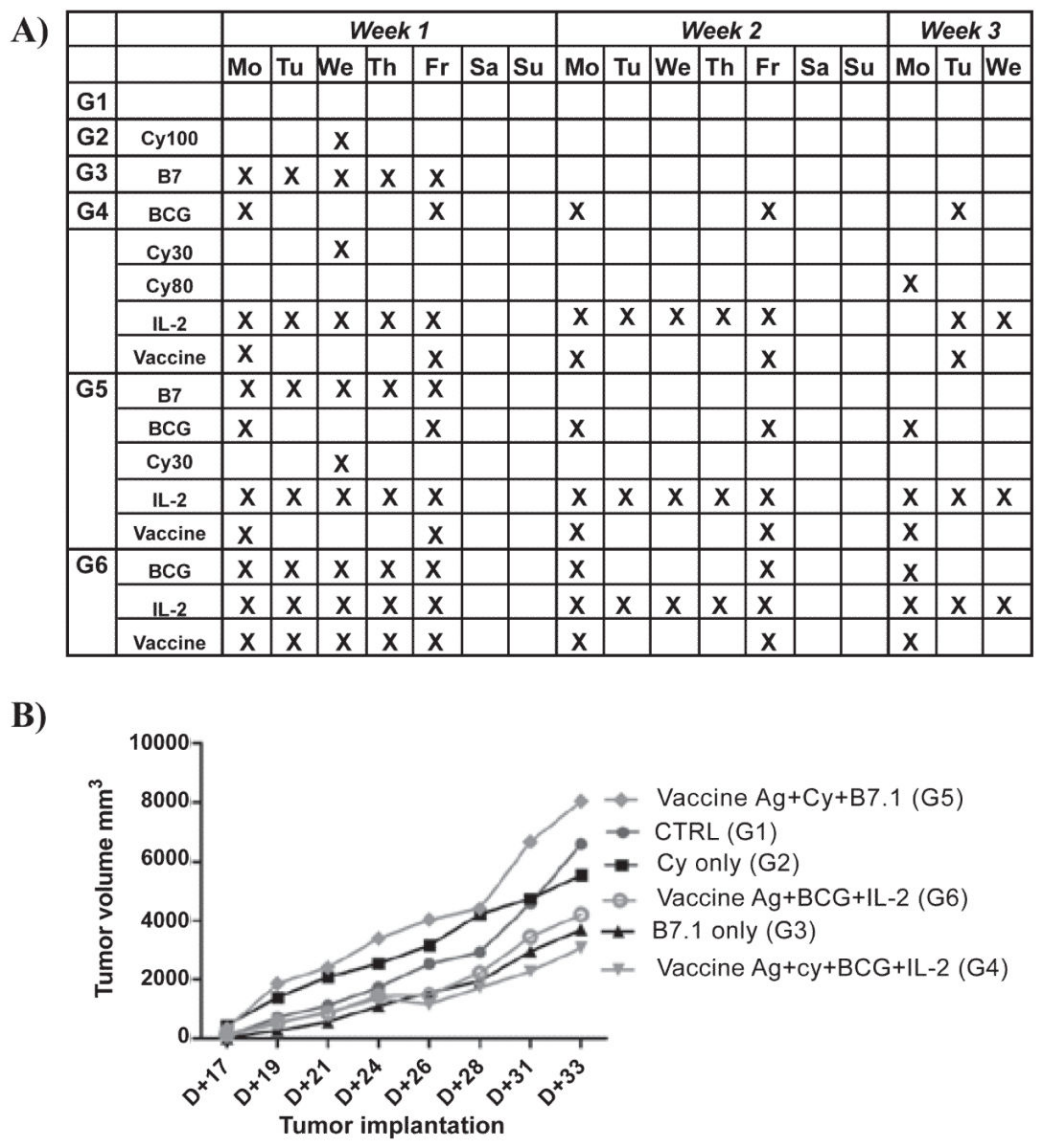
A vaccine was prepared from allogeneic and syngeneic antigens and administered in a fractionated regimen together with BCG and IL-2 to determine the effects on CNS-1 glioma growth A) Treatment schedule representing one cycle of the various immunotherapeutic injections. B) Average values of tumor growth as a function of time post-implantation for the different treatment groups.

**Figure 2:**
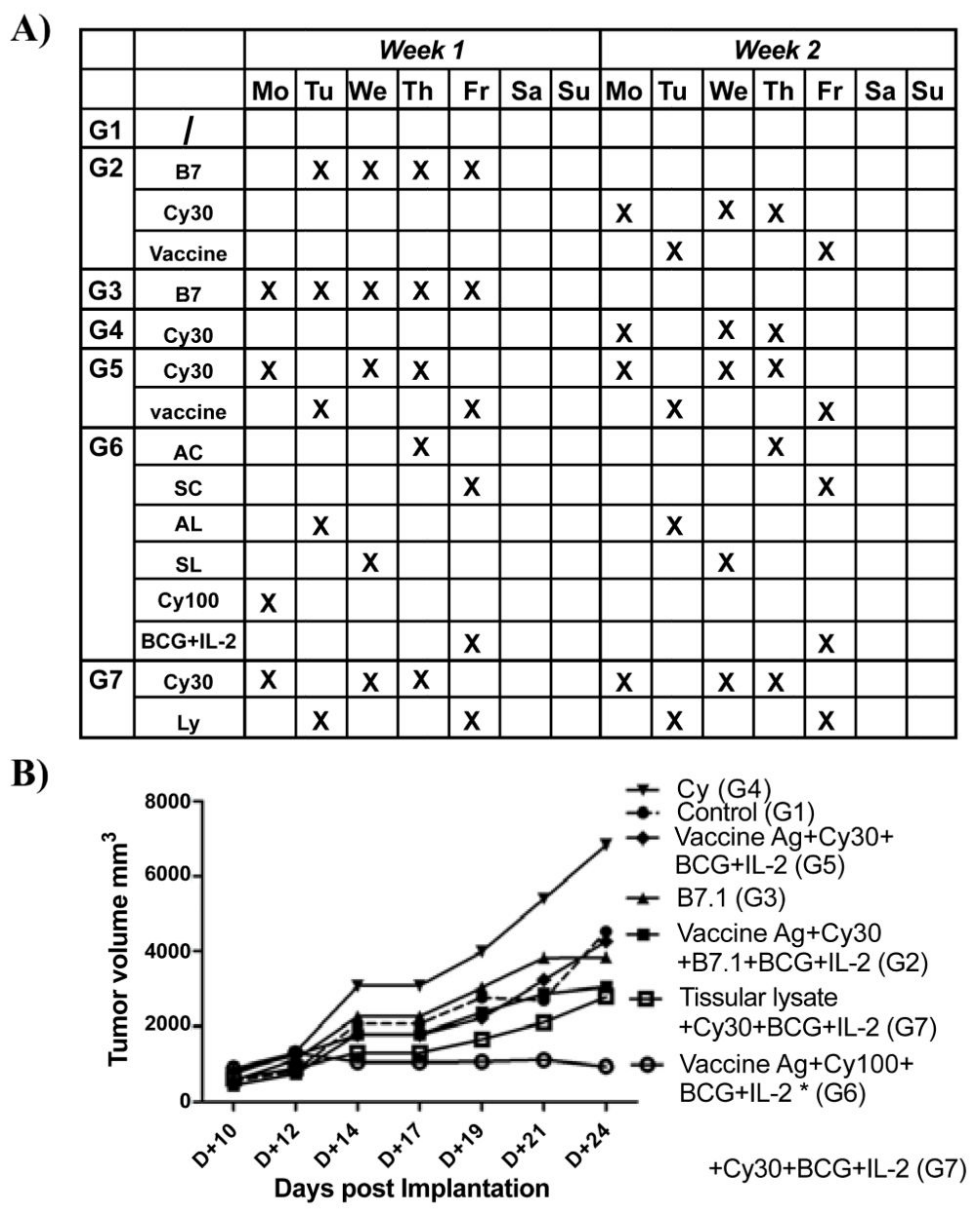
A vaccine was prepared from allogeneic and syngeneic cells administered in a fractionated regimen together with BCG and IL-2 A) Treatment schedule representing the cycle of the various immunotherapeutic injections. AC, allogenic cells, SC, syngeneic cells, AL, allogeneic lysate, SL, syngeneic lysate, Ly, is lysate only. B) Average values of tumor growth as a function of time post-implantation for the different treatment groups.

This vaccine preparation was kept as a constant factor and given in combination with various other anti-tumor or immunostimulatory agents as specifed in Figures 1A & 2A. The TLR7/8 agonist resiquimod was co-administered 3 times a week on Mondays, Wednesdays and Fridays.

## Chemicals and Reagents

### Immunomodulators and potentiators

Rats were subcutaneously (SC) injected in the flank, contralateral to the tumor-implanted side, with resiquimod (R848) (Invitrogen, TLRL-R848), a Toll-like receptor 7/8 agonist, at a dose of 100 μg/kg, corresponding to 30 μg/dose, three times per week on Mondays, Wednesdays and Fridays.

In a parallel arm of the experiment we evaluated the effect of cyclophosphamide (CY) administration, in diverse regimens and dosing as specified, on CNS-1 glioma development. Cyclophosphamide (CalBiochem, 239785) was given at 30 mg/kg 3 times per week, or at 100 mg/kg once every week (See also Figure 1A and 2A).

We also tested the vaccine antigen preparations combined with Bacilles Calmette-Guérin, interleukin-2 (IL-2), or a B7.1 fusion protein.

The Bacilles Calmette-Guérin (BCG), substrain Connaught, (Immunocyst®, Sanofi Pasteur) is an agonist of TLRs 2, -4 -9, and was used at 2 × 10^e^5 CFU per dose.

Interleukin-2 (IL-2) was injected daily at a dose of 75,000 IU/day from Monday to Friday starting on the day of vaccine injection.

A B7.1 fusion protein consisting of the extracellular domains of human B7.1 and the Fc portion of human IgG1, called B7.1-Fc, was produced as described [8]. This protein induced complete regression of Colon 26 tumors in a mouse model and slowed tumor growth dramatically in mice with established poorly immunogenic RENCA and Madison109 tumors [8]. The B7.1-Fc protein was diluted in sterile PBS at 250 μg/200μl concentration.

For statistical analysis, we used ANOVA nonparametric testing followed by student’s *t* tests to compare groups. P values of < 0.05 (*), p < 0.01 (**), p < 0.001 (***) were considered statistically significant.

## Results

### Efficacy of a BCG-containing vaccine administered at day 17 after implantation

CNS-1 tumor cells (2 × 10^5^ cells / 200 μl) were implanted in syngeneic male Lewis rats and after 17 days were either left untreated (control group; n = 4) or administered with a therapeutic immunomodulatory regime or some of its components, as specified in the treatment schedule of Figure 1A.

**Figure 3:**
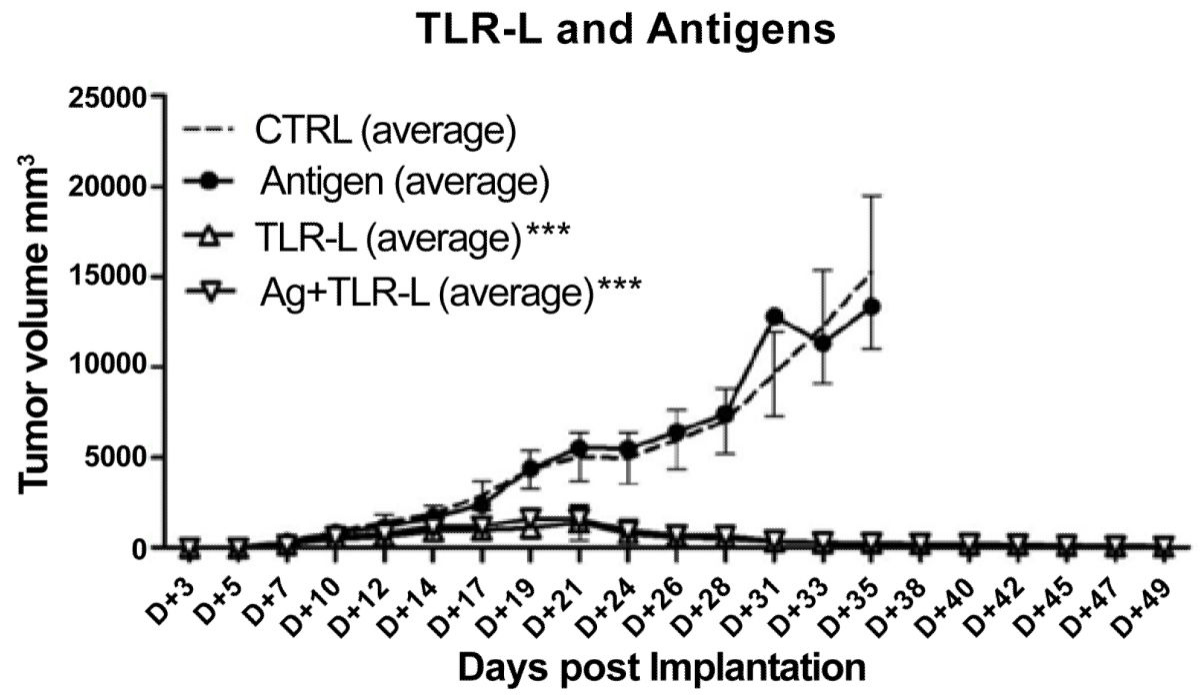
A vaccine was prepared from the standardized allogeneic and syngeneic cells administered with or without the TLR7/8 agonist, resiquimod, injected 3 times a week on Mondays, Wednesdays and Fridays. Average values of tumor growth as a function of time post-implantation for the different treatment groups.

The vaccine antigen preparation consisted of a mixture of haptenized 10^e^6 syngeneic and 10^e^6 allogenic RG2 glioma cells together with lysates produced from 3 × 10^e^6 synegeic CNS-1 cells and 3 × 10^e^6 allogenic RG2 gliomacells (n = 4). When monitoring tumor growth over time with a caliper, we observed no significant difference in tumor volumes between the control group (G1) and the rats receiving cyclophosphamide (CY) only at a dose of 100 mg/kg (G2) (Figure 1). From other experiments we had learned that the vaccine antigen preparation showed no anti-tumor efficacy by itself under the conditions described (for illustration see also figure 3, the antigen only group indicated by closed circles). By contrast, when this vaccine antigen preparation was administered together with the multiple TLR agonist BCG plus IL-2, an inhibition of tumor growth was noted (G6). Similarly, the same treatment schedule supplemented with CY (30 mg/kg on day 19, and 80 mg/kg on day 31) also inhibited tumor growth (G4). Remarkably, also five daily injections of B7.1-FC fusion protein alone (Monday to Friday, starting day 17 post implantation), showed some inhibition of tumor growth (G3), though not statistically significant relative to control groups. However, when combining the beneficial vaccine preparation plus CY (30 mg/kg) treatment with an additional regimen of B7.1-Fc antibody injections no inhibition of tumor growth was noted (G5).

### A BCG-containing vaccine shows better efficacy amongst the diverse vaccine prototypes tested

In a subsequent experiment, CNS-1 tumor cells (2 × 10^5^ cells / 200 μl) were implanted in 8-12 week-old syngeneic male Lewis rats and were either left untreated (control group; n = 4) or administered with a therapeutic immunomodulatory treatment regimen or some of its components, as specified in the treatment schedule depicted in Figure 2A. Instead of waiting until day 17 post tumor implantation, we now started at day 10 after tumor inoculation.

As before, the vaccine antigen preparation consisted of a mixture of haptenized syngeneic CNS-1 and allogeneic RG2 glioma cells (10^e^6 each) with lysates produced from syngeneic CNS-1 cells and allogeneic RG2 glioma cells (3 × 10^e^6 each) (n = 4). When monitoring tumor growth over time with a caliper, we observed no significant difference in tumor volumes between the untreated control group (G1) and the rats receiving low dose cyclophosphamide (CY, 30 μg/dose) in week two (G4), B7.1-Fc fusion protein only (G3), B7.1-Fc protein plus CY (G2) or vaccine plus CY only (G5) (Figure 2A). By contrast, when this vaccine antigen preparation was administered together with the multiple TLR agonist BCG plus IL-2 and CY (100 μg/dose), a significant inhibition (p < 0.05) of tumor growth was noted (G6). This vaccine preparation was administered in a fractionated schedule starting with allogeneiic cells on the first day, followed by syngeneic cells the second day, and allogeneic and syngeneic lystates on the third and fourth days, respectively (G6). Remarkably, also *ex vivo* tumor tissue isolated from a syngeneic established CNS-1 tumor plus CY (G7) showed some inhibition of tumor growth (although not statistically significant) relative to control groups (Figure 2B).

### Therapeutic treatment with a vaccine containing immunostimulatory TLR7/8 agonist shows marked inhibition of glioma tumor growth

In view of the beneficial effect of vaccination in the context of the multi-TLR agonist BGC, we decided to evaluate the effect of a TLR7/8 agonist in our CNS-1 tumor model. CNS-1 tumor cells (2 × 10^5^ cells/200 μl) were implanted in 8-12 week-old syngeneic male Lewis rats and either left untreated (control (CTRL) group; n = 8) or treated at day 10 after implantation with a vaccine antigen preparation consisting of a mixture of haptenized syngeneic CNS-1 and allogeneic RG2 glioma cells (10^e^6 each) with lysates produced from syngeneic CNS-1 and allogeneic RG2 glioma cells (3 × 10^e^6 each) (n = 8), given alone or combined with the TLR7/8 agonist, resiquimod (R848). This vaccine preparation was given 3 times a week on Mondays, Wednesdays and Fridays. When monitoring tumor growth over time with a caliper, we observed no significant difference in tumor growth between control and vaccine antigen only treated rats (Figure 3). By contrast, when this antigen preparation was administered together with a TLR7/8 agonist (30 μg/dose) significant inhibition (p, 0.001, ***) of tumor growth, with complete tumor regression was noted (Figure 3). Remarkably, administration of TLR7/8 agonist alone in the same regimen also inhibited tumor (p, 0.001, ***) (Figure 3).

### Inhibition of tumor progression with large established glioma tumors by a vaccine containing the TLR7/8 immunostimulant

**Figure 4:**
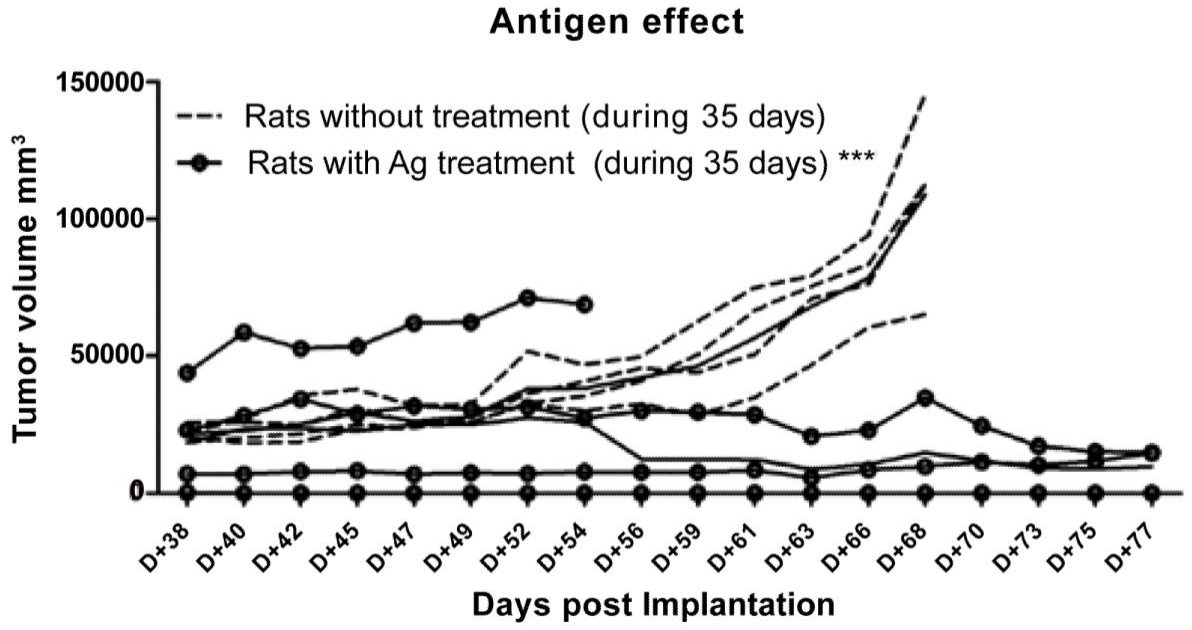
Individual tumor growth curves as a function of time post-implantation.

As mentioned above, Lewis rats treated with a vaccine without immunostimulatory TLR7/8 agonist showed little inhibition of tumor growth relative to untreated control rats, while the TLR7/8 containing vaccine strongly suppressed tumor growth when given 3 times per week starting at day 10 after implantation. We therefore decided to examine the effect of therapeutic vaccination of animals which showed no inhibition of tumor growth at day 34 after implantation. We administered the TLR7/8 containing vaccine to animals with large (12-14,000 mm^3^) tumors, starting at 38 days after tumor implantation. The TLR7/8 agonist-containing vaccine was injected 3 times a week on Mondays, Wednesdays and Fridays, in both untreated control rats (n=4) and rats immunized with idential antigen only (n=4). We noted in both groups a slower rate of growth of gliomas with large tumor volumes, but the TLR7/8 containing vaccine was best able to evoke a significant (p < 0.001, ***), arrest of tumor volume growth in the rats that were treated before with the antigen only vaccine preparation, relative to rats which were untreated (Figure 4). As well, no signs of vaccination-induced adverse effects or toxicity were observed, confirming data from another study that immunotherapy is well tolerated with limited toxicity [9]. These untoward effects point to the relative safety and tolerability of vaccines.

## Discussion

In the present study we show that a prototype vaccine, consisting of a mixture of allogeneic and syngeneic glioma cells and their lysates, administered together with either a multiple TLR2, -4 and -9 agonist BCG [9], or the TLR7/8 agonist resiquimod, is able to inhibit CNS-1 glioma brain tumor growth in syngeneic Lewis rats. For early stage tumors, a complete regression of tumor growth volume was noted, while for large established 38-day old tumors (14,000 mm^3^ volume), inhibition of tumor growth was noted for the TLR7/8 containing vaccine. These data confirm the well-know phenomenon that large glioma tumors are more difficult to control relative to smaller tumors. Nevertheless, if the latter is most relevant to the large tumor burden that may be present in individuals with gliomas in unresectable sites (e.g. parietal or tempero-parietal tumors), instituting vaccination with TLR agonists may be valuable in providing additional quality survival time.

During glioma tumor development a proportional increase in local and systemic immunosuppression has been documented [6,9,11,12]. Gliomas are known to contain T regulatory cells, which normally act as suppressor cells, regulating homeostatis and preventing autoimmune reactions. However, regulatory T cells also inhibit T effector functions, activated naturally by vaccination [13,14], and thereby facilitating tumor immune evasion and subsequent tumor progression. At the molecular level this is associated with an increase in production of immune inhibitory cytokines including transforming growth fcator (TGF)-beta, and/or interleukin-10 [15]. In general, immunosuppression can be counteracted by either strong stimulation of proinflammatory immune pathways, by blocking immune inhibitory elements triggered by the tumor itself, or by a combined “push-pull” approach [16]. We therefore evaluated well-known costimulation-enhancing immunostimulants, such as TLR-agonists [17], and inhibitors of immune system signals, such as what the B7.1 and cyclophosphamide immunomodulators provide. They were administered in conjunction with our standard vaccine antigen preparation.

We noted beneficial antitumor activity with daily injection of the B7.1Fc fusion protein starting at day 17 after tumor implantation as a monotherapy. This soluble costimulator protein was fused to the Fc portion of the antibody. It has been developed and tested for the immunotherapy of solid tumors in mouse tumor models [8]. This B7.1-Fc protein was found to be capable of activating T cells in vitro and in vivo [8]. B7.1 is able to engage with two known receptors. CD28, which triggers a stimulatory signal to activate naive T cells after binding by B7.1 (4), and its counterreceptor, CTLA-4, which triggers a negative signal to stop T-cell activation. Since B7.1 has high affinity to CTLA-4, soluble B7.1-Fc may block CTLA-4 signaling instead of cross-linking CTLA-4, thereby sustaining the activation of tumor-specific T cells [18,19]. Crosslinking of T cell activating CD28 may be less important than blocking the inhibitory CTLA-4, since activated T cells recognizing tumor antigens require less costimulation [20]. When B7.1 antibody was combined with other immune system activating treatments, we observed no synergistic activities, which illustrates that the timing and choice of modalities during combined immunotherapy is criticical for a beneficial antitumor effect.

Also, cyclophosphamide, when dosed carefully, has been demonstrated to facilitate immunotherapy of established tumors through the elimination/inactivation of suppressor T cells [21-23]. In our investigations, a protocol of cyclophosphamide (CY) only, administered at a dose of 100 mg/kg given on day 19 post transplantation did not affect tumor growth (Figure 1), while three lower doses of 30 mg/kg each starting on day 17 given within a week period, did not show a beneficial anti-tumor activity (Figure 2). By contrast, CY treatment alone may even exacerbate tumor growth, relative to the untreated control group, which may likely result from suppression of endogenous anti-tumor immune reactions in parallel to inadeqaute direct tumor growth inhibition, resulting in a net increase in tumor growth volume. Nevertheless, when combined with other immune adjuvants or vaccines, beneficial effects were noted despite co-administration of CY.

The best efficacy was noted in treatment schedules containing the vaccine antigen preparation plus BCG and IL-2. Bacillus Calmette-Guérin (BCG) is a mycobacterium which contains various agonists of Toll-like receptor 2 (TLR2) and TLR4 in its cell wall skeleton (CWS), and also BCG DNA which acts as a TLR9 agonist [10,24]. Intravesical immunotherapy with BCG, as monotherapy, causes significant reductions in cancer tumor progression and is currently the standard treatment for superficial bladder cancer [25]. However, the exact mode of action of successful BCG therapy remains elusive, although massive cytokine expression and influx of innate immune cells have been documented, which may be involved in tumor elimination.

Apart from BCG, we also examined the effect of the TLR7/8 agonist resiquimod as an adjuvant in a separate animal study. TLR7/8 agonists exert pleiotropic effects on various immune cells which leads to stimulation of proinflammatory cytokine and chemokine production, as well as up-regulation of costimulatory molecules on antigen presenting cells [26]. The TLR7/8 agonist resiquimod has been shown to act as a vaccine adjuvant in investigational vaccine models [25,27], and to generate clinical-grade mature DCs [28].

In our CNS-1 model, the TLR7/8 agonist resiquimod showed superior antitumor effects against early tumor growth and remarkably, administration of TLR7/8 agonist alone inhibited tumor growth. The exact mode of action for the observed resiquimod-mediated anti-tumor immunity needs to be defined in detailed follow-up studies. Most likely the observed resiquimod-based immunotherapy, even in absence of additional tumor antigens, is able to activate a spontaneous, natural, innate anti-tumor immune response, that under normal circumstances is unable to control tumor growth. Hence, such dedicated follow-up studies need to address to involvement of anti-tumor killer macrophages or NK cells, or IFNs for the resiquimod-induced glioma growth regression. In addition, the TLR7/8 vaccine preparation evoked an arrest of tumor volume growth in rats carrying large tumors that were treated before with the antigen only vaccine preparation.

Although the presented results are promising the should always be interpretaed with caution. Subcutaneous tumors may be exposed to markedly distinct immune conditions than in the brain, and so the physiologic as well as clinical relevance has to be validated in further studies.

Our vaccine preparation contains multiple tumor associated antigens (TAA) prepared from syngeneic and allogeneic cells. Although the precise identity of antigens is unknown we chose this antigen preparation for our vaccination strategy, rather than one or a few molecular targets, in order to target multiple TAAs simultaneously. This approach minimizes the chances for classical tumor-escape of immune control, as a result of selective growth of antigen-loss variants. With this approach we guaranteed that multiple relevant TAAs gain enhanced exposure to the immune system, while the self-antigens in this mixture are neglected as a result of immunological tolerance. By including cell associated antigens, we increased the chance of cross-presentation by MHC class I molecules for priming of cytolytic CD8^+^ T cells. By including TAAs of allogeneic origin, we additionally trigger allogeneic immune reactions. The haplotypes of CNS-1 and RG2 are fairly close, i.e., RT1^l^ vs RT1^lvl^ thus providing a mismatch that may be considered abberant self, and also induce a vigorous alloresponse. Allogeneic (major histocompatibility complex MHC-mismatched) tumor cells have shown better protection in a number of models likely due to the “non-self” immune recognition of these antigens [29-31].

Immunotherapy against TAA theoretically carries the risk of autoimmune reactions. Autoimmunity is possible, especially in view of our manipulations aimed at blocking immune response inhibitors coupled with the use of immunostimulating adjuvants. In the present studies, however, we have not observed any adverse reactions using our animal well-being scoring system. These observations are in line with well-documented observations, that therapeutic tumor vaccination is, in general, well-tolerized with minimal or no adverse events.

In conclusion, we noted beneficial antitumor activity of daily injection of the B7.1-Fc fusion protein as monotherapy, and with vaccine preparations that were administered with BCG and IL-2 only, or with the TLR7/8 agonist resiquimod. Altogether these data illustrate the importance of a beneficial immunomodulatory protocol. Different immunomodulators may result in distinct antitumor efficacy. Hence, we conclude from our data that only specific combinations of the right antigenic mixtures in conjunction with a suitable immunopotentiator is able to arrest aggressive glioma tumor growth in this experimental tumor model. Our data highlight the need for continued, more extensive exploration into such combinations.
